# Nanostructured Lipid Carriers as Physicochemical Modulators of Complex Natural Extracts: Release Behavior and Bile-Induced Remodeling in Biorelevant Media

**DOI:** 10.3390/molecules31061028

**Published:** 2026-03-19

**Authors:** Javiera Carrasco-Rojas, Javiera Solas-Soto, Rubén Veas-Albornoz, Carlos F. Lagos, Mario J. Simirgiotis, Francisco Arriagada, Andrea C. Ortiz

**Affiliations:** 1 Departamento de Ciencias y Tecnología Farmacéutica, Facultad de Ciencias Químicas y Farmacéuticas, Universidad de Chile, Santiago 8380494, Chile; javiera.carrasco@uchile.cl (J.C.-R.); francisco.arriagada@ciq.uchile.cl (F.A.); 2 Escuela de Química y Farmacia, Facultad de Ciencias, Universidad San Sebastián, Lota 2465, Santiago 7510157, Chile; jsolass@correo.uss.cl (J.S.-S.); ruben.veas@uss.cl (R.V.-A.); carlos.lagos@uss.cl (C.F.L.); 3 Centro Basal Ciencia & Vida, Fundación Ciencia & Vida, Av. del Valle Norte 725, Santiago 8580702, Chile; 4 Instituto de Farmacia, Facultad de Ciencias, Universidad Austral de Chile, Campus Isla Teja, Valdivia 5090000, Chile; mario.simirgiotis@uach.cl

**Keywords:** nanostructured lipid carriers, propolis, biorelevant gastrointestinal media, HepG2

## Abstract

Propolis is a chemically complex natural product with recognized antioxidant potential, but its compositional heterogeneity and poor aqueous solubility complicate formulation and interpretation of in vitro release behavior. In this study, a nanostructured lipid carrier (NLC) based on Gelucire^®^ 44/14 was developed as a physicochemical platform to modulate the accessibility of a selected Chilean ethanolic propolis extract. Propolis extracts from different origins were first screened using complementary antioxidant assays (DPPH, ABTS, ORAC, FRAP), leading to the selection of the Peñaflor extract, which exhibited the highest phenolic content (~41 mg GAE/g) and antioxidant capacity. The selected extract was incorporated into NLCs with encapsulation efficiencies above 90%, a narrow size distribution (~200 nm), and high stability over 90 days. Under simple aqueous conditions, propolis release remained limited (<15% over 6 h), consistent with diffusion- and partition-controlled transport. In simulated gastrointestinal media containing bile components, pronounced pH- and composition-dependent effects were observed. While fed-state intestinal conditions induced extensive morphological remodeling without increasing the analytically accessible fraction (<3% at 4 h), fasted-state intestinal media promoted a higher accessible fraction (~14% within 1 h) without complete carrier disruption, as confirmed by transmission electron microscopy. Preliminary cytocompatibility studies in HepG2 cells showed acceptable viability at 10–40 µg/mL and concentration-dependent effects at higher doses. Overall, this work demonstrates that bile components modulate propolis accessibility through dynamic partitioning and colloidal reorganization rather than simple carrier breakdown, providing a physicochemical framework for future digestion and absorption studies.

## 1. Introduction

Propolis is a chemically complex resinous material produced by bees through the transformation of plant-derived exudates, and it is widely recognized for its rich content of polyphenols, flavonoids, and other redox-active constituents [1]. Owing to this composition, propolis has been associated with antioxidant, antimicrobial, and anti-inflammatory properties, motivating sustained interest in pharmaceutical and nutraceutical research [2,3]. However, propolis does not represent a single, well-defined chemical entity; its composition is highly heterogeneous and strongly dependent on botanical source, geographic origin, and local environmental conditions, leading to pronounced variability even among samples collected within geographically close regions [4,5]. This intrinsic heterogeneity poses challenges for formulation development and complicates the interpretation of in vitro performance data.

From a physicochemical standpoint, additional limitations arise from the poor aqueous solubility and marked lipophilicity of many propolis constituents [6]. Under gastrointestinal conditions, these properties may restrict the fraction of compounds that become analytically accessible, resulting in low apparent release or activity values that reflect formulation-dependent partitioning phenomena rather than insufficient intrinsic chemical potential [7,8]. Consequently, conventional in vitro assays may underestimate or misrepresent the functional behavior of propolis if the formulation context, phase behavior, and analytical readouts are not carefully considered.

Several delivery systems have been explored to improve the stability and bioavailability of propolis and other polyphenol-rich natural extracts, including liposomes, polymeric particles, and solid lipid nanoparticles (SLNs) [9]. Liposomal systems are attractive due to their biocompatibility and ability to encapsulate both hydrophilic and lipophilic compounds [6]; however, their phospholipid bilayers may be destabilized under gastrointestinal conditions, particularly in the presence of bile salts and digestive surfactants, leading to membrane disruption and premature compound leakage [10]. Polymeric carriers such as PLGA-based microspheres can provide controlled release and protection against degradation, but their preparation typically involves multistep processes and organic solvents, which may complicate large-scale production and limit encapsulation efficiency for chemically heterogeneous extracts. In addition, their degradation behavior and release kinetics in the digestive environment may be difficult to predict [11,12]. Conventional SLNs have also been investigated for natural product delivery; however, their highly ordered lipid matrices often restrict drug accommodation and may promote compound expulsion during lipid crystallization, particularly when accommodating complex lipophilic mixtures [13].

In this context, nanostructured lipid carriers (NLCs) have emerged as versatile colloidal systems for the incorporation of poorly water-soluble compounds and complex natural extracts [14]. By combining solid and liquid lipid components, NLCs form partially disordered matrices capable of accommodating high payloads while maintaining colloidal stability [15]. For chemically heterogeneous natural extracts such as propolis, where multiple constituents with different polarity and partitioning behavior coexist, this structural characteristic is particularly advantageous because it enables the incorporation of diverse lipophilic constituents while minimizing crystallization-induced expulsion phenomena [16]. Importantly, NLCs should not be regarded solely as passive encapsulation vehicles. Their internal organization, interfacial properties, and interactions with surrounding media govern not only loading efficiency and physical stability but also the fraction of encapsulated compounds that become accessible under specific physicochemical environments [17]. In this sense, lipid-based nanocarriers can actively modulate compound partitioning, apparent release, and analytical accessibility, particularly in complex biorelevant media.

For chemically heterogeneous systems such as propolis, rational extract selection represents a critical initial step. Antioxidant profiling, when interpreted as a physicochemical descriptor rather than a direct predictor of biological efficacy, provides a comparative chemical framework to guide this selection. The use of complementary antioxidant assays offers an integrated view of redox-related properties and reduces the risk of advancing extracts whose apparent activity is dominated by assay-specific effects or narrow subsets of reactive constituents [18]. This strategy is especially relevant when the objective is to investigate how a lipid-based nanosystem modulates accessibility and release behavior, rather than to rank extracts solely on the basis of bioactivity claims.

In parallel, the gastrointestinal environment introduces additional complexity for lipid-based nanocarriers. Bile salts and phospholipids are surface-active amphiphiles capable of inducing interfacial remodeling, mixed colloid formation, and redistribution of lipophilic compounds [19]. Importantly, structural alteration of lipid carriers in bile-containing media does not necessarily translate into proportional increases in analytically measured release, as morphological changes and assay-accessible fractions represent distinct physicochemical readouts. Understanding this distinction is essential for the rational interpretation of release data obtained in biorelevant media.

Unlike most previous studies on propolis delivery systems, which primarily focus on improving encapsulation efficiency or biological activity, the present work investigates how a lipid nanosystem modulates the physicochemical accessibility and apparent release of a chemically complex extract under biorelevant gastrointestinal conditions. Specifically, this study aimed to develop and characterize a Gelucire^®^-based nanostructured lipid carrier loaded with a selected Chilean propolis extract from the Metropolitan Region and to investigate how simulated gastrointestinal environments containing bile components influence carrier structure and apparent propolis release. We hypothesized that the nanostructured lipid carrier would behave as a retentive lipid matrix under simple aqueous buffer conditions, while bile salts and phospholipids present in biorelevant media would induce composition- and pH-dependent interfacial remodeling and redistribution of propolis into mixed colloidal assemblies. Such processes were expected to alter the assay-accessible fraction without necessarily implying complete carrier disintegration. To address these questions, propolis extracts from different local origins were first screened using complementary antioxidant assays to support rational extract selection. The selected extract was subsequently incorporated into NLCs, followed by systematic evaluation of loading efficiency, colloidal properties, and physical stability. Release behavior was assessed in aqueous buffers and bile-component-containing simulated gastrointestinal media, alongside morphological analysis by transmission electron microscopy. Finally, a preliminary cytocompatibility assessment in HepG2 cells was conducted to support the potential relevance of the developed nanosystem for future biomedical applications.

## 2. Results and Discussion

### 2.1. Antioxidant Profile of Propolis Extracts as a Physicochemical Criterion for Nanosystem Design

The antioxidant characterization of the ethanolic propolis extracts (EPE) was conducted as an initial physicochemical screening step to support the rational selection of the extract to be incorporated into the NLC. Given the well-known compositional heterogeneity of propolis, the use of complementary antioxidant assays provides an integrated chemical description of its redox-related properties, rather than a direct prediction of biological efficacy [20]. In this context, antioxidant profiling was intentionally employed as a comparative physicochemical descriptor to guide formulation decisions while minimizing assay-specific bias. The results obtained for total phenolic content (TPC) and antioxidant assays are summarized in Table 1. TPC analysis revealed marked quantitative differences among the extracts, with the Peñaflor sample exhibiting the highest phenolic content (~41 mg GAE/g extract), followed by Pirque (~37 mg GAE/g extract) and Pudahuel (~35 mg GAE/g extract). These differences are consistent with the strong influence of botanical origin and local vegetation on the chemical fingerprint of Chilean propolis, even within geographically close areas of the Metropolitan Region [21]. From a physicochemical perspective, higher TPC values indicate a greater density of redox-active moieties capable of participating in electron transfer and hydrogen atom donation processes [22]. However, it should be emphasized that TPC alone does not establish a linear or universal relationship with antioxidant performance, reinforcing the need for complementary functional assays.

Radical scavenging assays further supported this comparative trend. In both DPPH• and ABTS•^+^ assays, the Peñaflor propolis extract displayed the lowest SC50 values (53.4 µg/mL and 2.6 µg/mL, respectively), indicating higher apparent scavenging efficiency than the Pirque and Pudahuel extracts (Table 1). Although DPPH• and ABTS•^+^ are often grouped as conceptually similar assays, their distinct radical species, solvent environments, and reaction mechanisms provide complementary information [23]. The consistent ranking observed across both assays suggests that the superior performance of the Peñaflor extract is not an assay-dependent artifact but rather reflects a broader compositional advantage within the limits of chemical redox-based measurements.

Additional insight was provided by the ORAC and FRAP assays, which probe different antioxidant mechanisms. ORAC values, reflecting the ability to scavenge peroxyl radicals under kinetic conditions, were markedly higher for the Peñaflor extract (~867 µmol Trolox/g) than for Pirque (~472 µmol Trolox/g) and Pudahuel (~322 µmol Trolox/g), indicating a pronounced difference in the capacity to delay oxidative chain reactions over time. In contrast, FRAP values followed the same general ranking but exhibited a narrower quantitative separation among extracts (Table 1). Although these differences are modest, their consistency with the trends observed in TPC, DPPH•, ABTS•^+^, and ORAC supports their interpretation as part of a coherent multidimensional physicochemical profile rather than as isolated numerical descriptors. Such partial divergence among assays is expected for complex natural matrices and underscores the importance of interpreting antioxidant data collectively rather than relying on a single metric [24]. Importantly, the convergence of all applied assays toward the same extract as the most redox-active supports the robustness of the selection strategy. This robustness should be understood strictly within the framework of in vitro chemical characterization and does not imply predictive value for in vivo antioxidant efficacy. Rather than advancing an extract based on a single antioxidant endpoint, the combined evaluation of TPC, radical scavenging capacity and reducing power enables identification of the extract with the most balanced and consistent redox-related properties. From a formulation standpoint, this approach minimizes the risk of advancing an extract whose apparent antioxidant performance is driven by assay-specific effects or by a limited subset of reactive constituents [25]. Based on this integrated antioxidant profiling, the Peñaflor ethanolic propolis extract was selected as the most suitable candidate for incorporation into NLC system. This selection does not imply superior biological activity per se but instead identifies an extract with higher chemical reducing capacity and radical reactivity, properties that are particularly relevant when evaluating how a lipid-based nanosystem modulates payload accessibility, release behavior and apparent antioxidant response.

It is worth emphasizing that antioxidant assays performed directly on propolis-loaded NLC formulations do not provide a direct measure of the intrinsic antioxidant capacity of the encapsulated extract. In such dispersions, the observed response is governed by the fraction of phenolic compounds that are accessible to the reaction medium during the assay time scale, which may include surface-associated molecules or compounds released from the carrier [13]. Consequently, antioxidant measurements on NLC formulations were not used as selection criteria and are reported only as Supplementary Information (Table S1), as they primarily reflect the fraction of antioxidants accessible during the assay rather than the total antioxidant content of the encapsulated system. This approach avoids overinterpretation of formulation-dependent assay effects and ensures that both extract selection and nanosystem design remain grounded in sound physicochemical reasoning while explicitly acknowledging the methodological limitations of antioxidant screening assays.

### 2.2. Rational Selection of Propolis Loading

**Figure 1:**
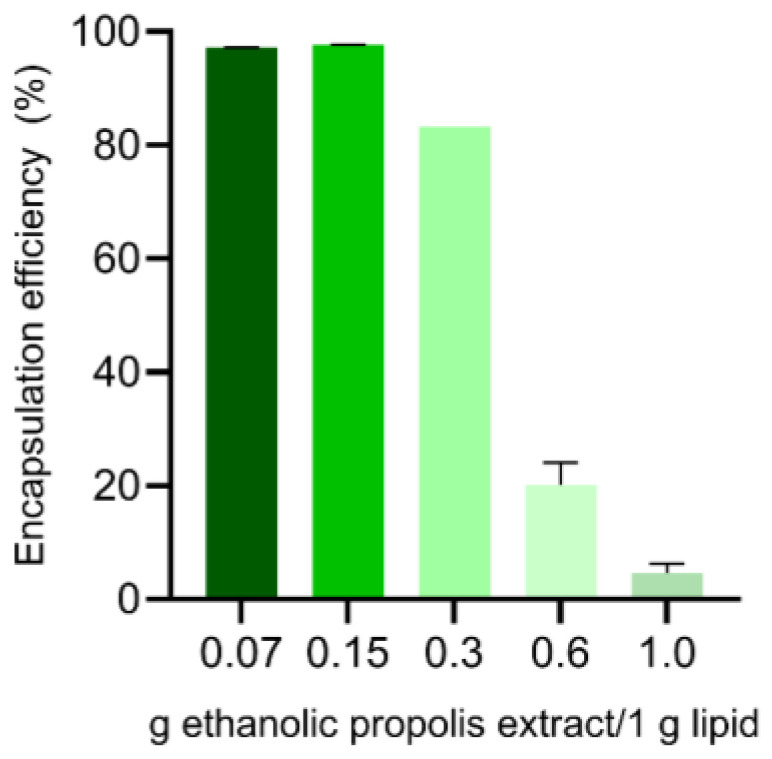
Encapsulation efficiency of ethanolic propolis extract from Peñaflor in the NLC. *n* = 3.

Several propolis-to-lipid ratios (expressed as g propolis extract equivalents per g Gelucire^®^ 44/14 (main lipid material)) were systematically evaluated to identify a formulation window that maximized effective encapsulation rather than nominal loading (Figure 1). Increasing the propolis content beyond this window led to a pronounced reduction in encapsulation efficiency, with high nominal loadings (e.g., 0.6 g propolis extract equivalents/g lipid material) resulting in efficiencies of only ~20–25%. In contrast, a propolis loading of 0.15 g propolis extract equivalents/g lipid material consistently preserved encapsulation efficiencies above 90%. Increasing the concentration to 0.6 g ethanolic propolis extract per 1 g of lipid resulted in a sharp decline in propolis encapsulation efficiency. This behavior reflects the finite solubilization and accommodation capacity of the lipid matrix for chemically heterogeneous natural extracts and highlights the importance of distinguishing between nominal loading and physically retained payload.

Such behavior is consistent with the literature on NLC systems loaded with complex natural extracts, which typically report encapsulation efficiencies ranging from 60% to 80% as a practical compromise between loading and stability [26]. The comparatively high encapsulation efficiency achieved here can be rationalized by the physicochemical characteristics of Gelucire^®^ 44/14, which, despite being classified as a lipid, exhibits self-emulsifying behavior due to its amphiphilic composition. This property facilitates the incorporation of compounds spanning a broad lipophilicity range, including moderately polar polyphenolic constituents. In addition, the intrinsic structural disorder of NLCs, arising from the combination of solid and liquid lipid phases, generates multiple molecular imperfections and free volume within the lipid matrix. These structural features are known to favor the accommodation and retention of bioactive compounds relative to fully crystalline lipid systems. For propolis in particular, the predominance of lipophilic flavonoids and related phenolic compounds further promotes favorable partitioning into the lipid domains, enhancing entrapment and reducing expulsion during nanosystem formation and storage [27]. On this basis, a propolis loading of 0.15 g propolis extract equivalents/g lipid material was selected as a rational formulation entry point for subsequent physicochemical, release, and biorelevant media studies.

### 2.3. Characterization of NLC and NLC-Pe

The physicochemical properties of the unloaded NLC and propolis-loaded formulation (NLC–Pe) are summarized in Table 2. The unloaded NLC exhibited a hydrodynamic diameter of 150 ± 3 nm, which increased to 207 ± 2 nm upon incorporation of the ethanolic propolis extract. This size increase is consistent with successful incorporation of extract constituents within the lipid matrix and has been widely reported for NLC systems loaded with natural products [28,29].

In both formulations, the PdI remained below 0.15, indicating narrow size distributions and good colloidal homogeneity. Zeta potential values were moderately negative (−10.3 mV and −8.0 mV for the NLC and NLC-Pe, respectively). Although, the zeta potential varied after the incorporation of EPE, the magnitude of this change is not expected to compromise colloidal stability. Instead, colloidal stability in this system is primarily governed by steric stabilization provided by the poly (ethylene glycol) chains present in Gelucire^®^, which form a hydrated interfacial layer that limits particle-particle interactions even at relatively low electrostatic potentials.

**Figure 2:**
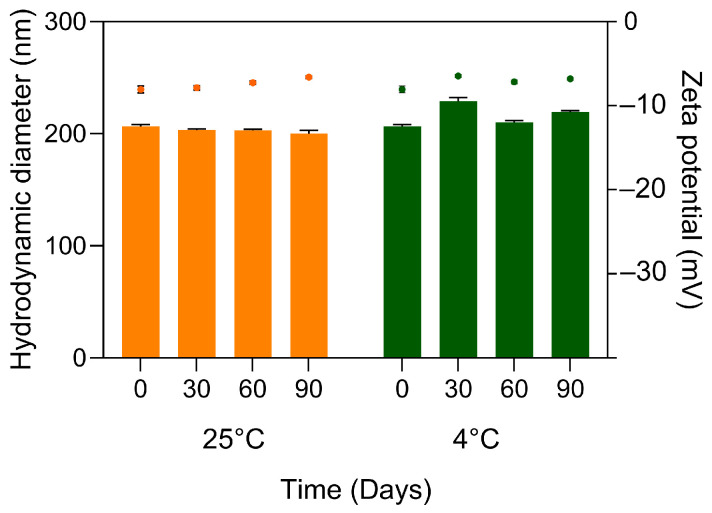
Colloidal stability of NLC and NLC-Pe over 0, 30, 60, and 90 days of storage at 25 °C and 4 °C (*n* = 3). Bar represents hydrodynamic diameter, while dots represent zeta potential.

Long-term colloidal stability studies further supported the robustness of the formulation. Over a storage period of 90 days at both 4 °C and 25 °C, no meaningful changes in hydrodynamic diameter, PdI, or zeta potential were observed (Figure 2). Importantly, encapsulation efficiency remained consistently above 96% at 30, 60, and 90 days, indicating minimal payload expulsion over time. Together, these results demonstrate that the selected propolis loading yields an NLC system with high physicochemical stability and retention capacity, suitable for subsequent evaluation under biorelevant release conditions.

### 2.4. Release Behavior in Aqueous Buffers

Because propolis is a chemically complex natural extract composed of numerous phenolic constituents rather than a single defined compound, release profiles were expressed as the cumulative mass of propolis extract equivalents (µg) detected in the external medium. This representation provides a direct estimate of the amount of extract that becomes analytically accessible under the tested conditions. Consequently, the reported values reflect the fraction of propolis that partitions into or becomes solubilized within the surrounding medium, rather than complete depletion of the extract from the lipid carrier.

**Figure 3:**
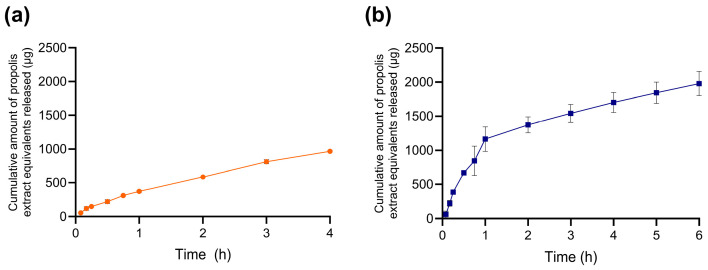
Apparent cumulative release profile of ethanolic propolis extract from NLC-Pe in aqueous buffers at (**a**) pH 2.0 and (**b**) pH 6.8. data are shown as mean ± SD (*n* = 3).

In acidic buffer (pH 2.0), NLC–Pe exhibited minimal release, reaching approximately 967 µg after 4 h, indicating strong payload retention under gastric-like aqueous conditions (Figure 3a). At pH 6.8, release followed a biphasic profile, with an initial increase to ~1100 µg within the first hour, followed by a slower phase reaching ~1900 µg after 6 h (Figure 3b). This change in slope is consistent with an initial readily accessible fraction (surface-associated or near-surface domains) followed by a slower transport process from deeper regions of the lipid matrix. Such behavior indicates that release under these simple aqueous conditions is governed primarily by diffusion and matrix-partitioning processes within the lipid carrier, rather than by aqueous pH alone. Importantly, the moderate difference observed between the two buffers is consistent with a retentive lipid matrix in which internal diffusion and lipid-extract interactions limit the direct influence of bulk pH on the analytically accessible fraction of propolis. These observations indicate that, under simple aqueous conditions, the nanosystem provides a suitable platform to examine how bile components modulate payload accessibility in more complex biorelevant media.

### 2.5. Effect of Bile-Component-Containing Media on Apparent Release and Morphology

**Figure 4:**
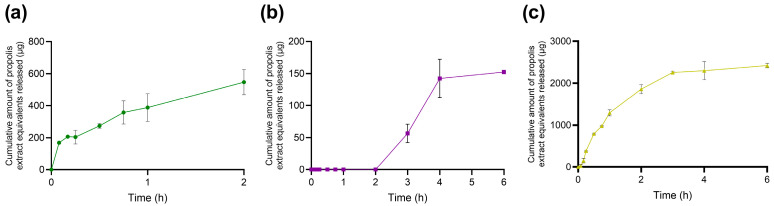
Apparent release profile of the ethanolic propolis extract from NLC-Pe in simulated gastrointestinal media containing bile components: (**a**) FaSSGF, (**b**) FeSSIF, and (**c**) FaSSIF. Data are shown as mean ± SD (*n* = 3).

**Figure 5:**
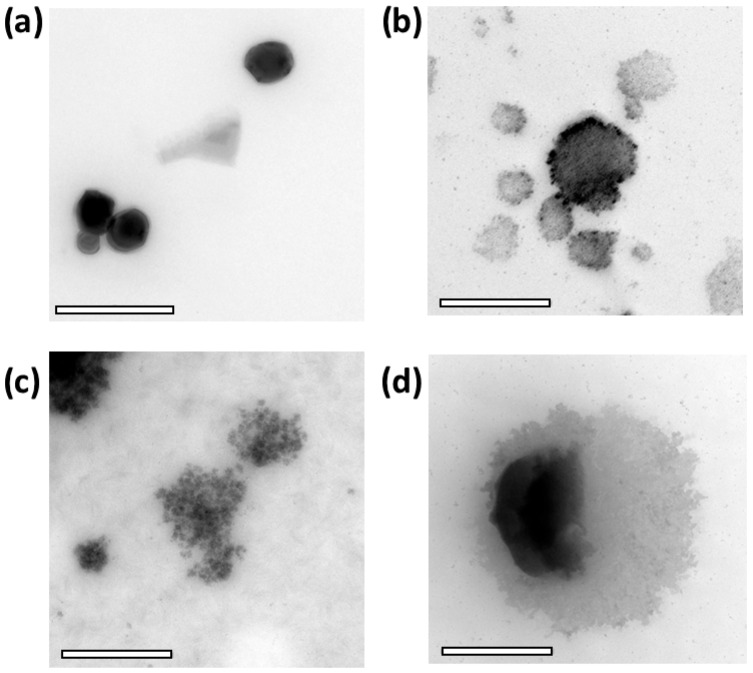
Transmission electron microscopy (TEM) images of (**a**) freshly prepared NLC-Pe (control), (**b**) NLC-Pe after incubation in FaSSGF, (**c**) NLC-Pe after incubation in FeSSIF, and (**d**) NLC-Pe after incubation in FaSSIF. All micrographs were acquired under identical imaging conditions at 20,000× magnification. Scale bar: 500 nm.

Bile salts are not passive solubilizers; they are highly surface-active amphiphiles capable of displacing interfacial layers, forming mixed micelles with phospholipids, and promoting lipid reorganization and solubilization processes [30]. Accordingly, the presence of bile salts and phospholipids markedly altered both the apparent release behavior and morphology of NLC-Pe in a pH- and composition-dependent manner. In the FaSSGF, which contains lecithin together with a low concentration of sodium taurocholate, apparent release remained low, reaching only ~550 µg after 2 h (Figure 4a), in line with limited propolis accessibility under strongly acidic conditions. Nevertheless, TEM images revealed increased morphological heterogeneity compared to freshly prepared NLC-Pe, including irregular and globular features (Figure 5b). These observations suggest bile-component-induced interfacial perturbation without extensive depletion of the propolis payload. Such behavior is consistent with previous reports showing that bile salts can induce permeability defects or partial structural rearrangements at sub-solubilizing conditions, producing visible morphological changes that do not necessarily translate into large increases in soluble payload during short time windows [31,32]. Therefore, under our conditions, this low micromolar taurocholate concentration is sufficient to perturb interfacial organization but remains far below levels typically associated with extensive lipid solubilization.

In FeSSIF, pronounced morphological remodeling was evident by TEM, including fragmentation and aggregation into smaller lipid domains (Figure 5d). Despite this extensive structural alteration, apparent propolis release remained very low, with little to no release detected initially and only ~150 µg after 6 h (Figure 4b). This apparent divergence between morphology and release is not unexpected in complex biorelevant media and can be rationalized by several non-mutually exclusive physicochemical factors. First, colloidal sequestration and phase partitioning are favored under FeSSIF conditions. Many phenolic constituents of propolis exhibit pH-dependent ionization behavior, and at mildly acidic pH they may remain less ionized and preferentially associated with hydrophobic or colloidal domains rather than partitioning into the assay-accessible aqueous phase. In this context, the high bile salt and phospholipid content of FeSSIF strongly promotes the formation of mixed lipid–bile assemblies that redistribute propolis within colloidal structures without necessarily increasing the analytically measurable fraction, as widely discussed for FaSSIF/FeSSIF media [33]. Second, the relatively high ionic strength characteristic of FeSSIF may contribute to aggregation-driven masking of release by compressing the electrical double layer and enhancing interparticle attraction, in line with classical DLVO considerations [34]. Such aggregation or bridging of remodeled lipid fragments can reduce the effective interfacial area available for net desorption of propolis markers into the sampled phase, while still yielding “damaged” or heterogeneous morphologies by TEM. Finally, it is important to emphasize that structural disruption and analytical accessibility represent fundamentally different readouts: TEM provides information on morphological states (e.g., fragments, aggregates, globules), whereas the release assay quantifies only the fraction that becomes accessible under the specific sampling and separation conditions employed. In bile-rich media, a substantial fraction of propolis may remain associated with lipid fragments or mixed colloids that are not fully represented in the quantified fraction, resulting in low apparent release despite pronounced morphological changes [31]. While these mechanisms cannot be directly disentangled within the scope of the present experiments, the combined release and TEM data support a coherent physicochemical framework for interpreting the observed behavior. Taken together, FeSSIF at pH 5.0 can be viewed as a condition that strongly remodels the carrier while simultaneously retaining or re-sequestering propolis within bile–phospholipid–lipid colloids.

In contrast, FaSSIF promoted a rapid increase in apparent release, reaching ~14% within the first hour, followed by a plateau at ~2400 µg up to 6 h (Figure 4c). This behavior is consistent with the rapid establishment of a partition equilibrium between the lipid matrix and bile component mixed colloids, yielding a larger assay-accessible fraction under fasted-state intestinal conditions. TEM images revealed altered particles surrounded by halo-like and globular features, indicative of lipid redistribution and secondary colloidal structures rather than complete carrier disintegration (Figure 5d). Notably, these results highlight that morphological alteration and apparent release are not linearly correlated in bile-component-containing media. In particular, FeSSIF induces extensive structural remodeling while retaining propolis within mixed colloidal assemblies, whereas FaSSIF favors higher analytical accessibility despite lower bile salt concentrations.

Although the simulated intestinal media used here did not include lipolytic enzymes, the observed remodeling is mechanistically relevant. Bile salts are known to condition lipid interfaces and facilitate subsequent lipase/colipase action by displacing interfacial material and organizing mixed assemblies [35,36]. Accordingly, the present results isolate the physicochemical contribution of bile components to carrier remodeling and payload accessibility. Fully dynamic digestion studies incorporating enzymatic lipolysis are expected to further modulate these processes and warrant dedicated investigation beyond the scope of the present work [35,37].

This overall interpretation is also consistent with prior studies demonstrating that bile salts can remodel lipid-based carriers without a one-to-one correspondence between structural alteration and the analytically recovered released fraction. In multiple lipid systems, taurocholate or cholate has been shown to trigger interfacial destabilization, vesicle–micelle transitions, or fragmentation under certain compositions and concentration windows, while substantial payload remains associated with mixed colloids or lipid remnants depending on pH and medium composition [31,36,38,39]. Collectively, these studies also emphasize that bile salts do not universally “destroy” lipid carriers in a single step; rather, they promote dynamic reorganization and partitioning processes whose analytical manifestation depends strongly on medium composition and the definition of the measured phase [40,41].

### 2.6. Assessment of Release Under Near-Physiological pH (pH 7.4)

**Figure 6:**
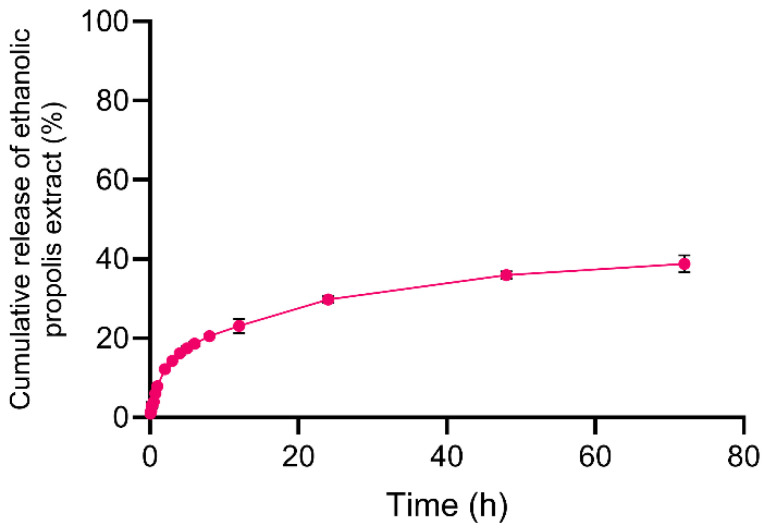
Apparent release profile of ethanolic propolis extract from NLC-Pe in buffer at pH 7.4. data are shown as mean ± SD (*n* = 3). Percentage release was calculated relative to the initial amount of propolis encapsulated in the NLC formulation.

To further explore release behavior once bile-driven perturbation is no longer dominant, NLC-Pe was evaluated in buffer at near-physiological pH (7.4). Under these conditions, release increased gradually from ~20% at 12 h to ~30% at 24 h, approaching ~40% after 72 h (Figure 6). This sustained release profile is consistent with diffusion- and partition-controlled transport from internal lipid domains over extended times scales. While this experiment does not model absorption or cellular uptake, it supports the premise that a substantial fraction of propolis remains kinetically retained within the lipid matrix and can be released under less aggressive aqueous conditions.

Comparable trends have been reported for other lipid-based nanosystems. Bidabad et al., observed approximately 20% release of propolis from liposomes after 48 h at pH 7.4 [42], whereas Aytekin et al. reported release values ranging from 40 to 80%, depending on propolis loading and formulation composition [43]. In hybrid systems, Bose et al., showed that increasing the amount and complexity of lipid layers around PLGA nanoparticles significantly delayed payload release compared to polymeric controls [44]. Taken together, these findings support the notion that lipid components can effectively slow the release of propolis constituents, likely reflecting their preferential partitioning into hydrophobic domains within the carrier matrix.

### 2.7. Cell Viability Assessment

Cell viability was evaluated as a preliminary cytocompatibility screening rather than a comprehensive toxicological assessment. The selection of the HepG2 cell line was based on its extensive use as an in vitro human hepatic model for nanocarrier cytocompatibility and toxicity screening, as well as its relevance for evaluating potential systemic toxicity of drug delivery systems. The liver represents the primary organ involved in xenobiotic metabolism and nanoparticle clearance, making hepatocyte-derived models particularly relevant for preliminary safety assessment of nanomaterials [45,46]. The tested concentration range was selected to enable comparison with previously reported propolis-based and lipid-based nanosystems evaluated under similar in vitro conditions. Accordingly, the results should be interpreted in the context of relative cytocompatibility and concentration—dependent trends rather than absolute toxicity thresholds [47,48].

**Figure 7:**
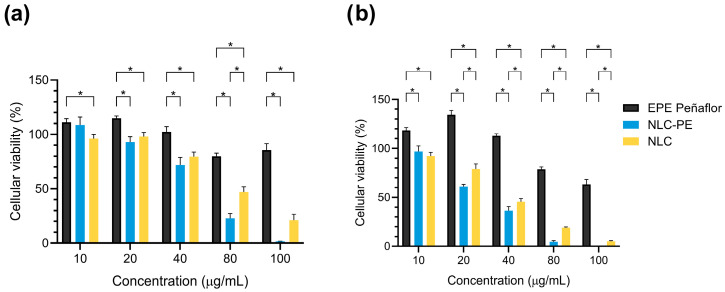
MTT-based cell viability of free ethanolic propolis extract, NLC, and NLC-Pe after (**a**) 24 h and (**b**) 48 h of exposure. * Indicates statistically significant differences. Data are shown as mean ± SD (*n* = 3).

Free ethanolic propolis extract from Peñaflor did not reduce HepG2 viability within the 10–40 µg/mL range, with cell viability remaining at or above 100% after both 24 and 48 h of exposure (Figure 7). This response is consistent with the antioxidant-rich composition of the extract and suggests the absence of acute cytotoxic effects at low to moderate concentrations under the tested conditions [48]. When the lipid carrier was evaluated independently (blank NLC), a moderate reduction in cell viability was observed at increasing concentrations, with values decreasing below ~80% from 40 µg/mL onward, suggesting that the lipid nanocarrier itself may contribute partially to the observed cytotoxicity at higher doses, likely through enhanced cellular interactions or membrane perturbation. In contrast, NLC-Pe formulations exhibited a clear concentration-dependent effect. High cell viability was maintained at 10 and 20 µg/mL, whereas progressive reduction was observed at higher concentrations. At 80 µg/mL, cell viability dropped to approximately 20–30%, and at 100 µg/mL a near-complete loss of viability was detected at both 24 and 48 h. The stronger reduction observed for NLC-Pe compared with blank NLC suggests that the incorporation of propolis into the lipid nanocarrier increases the effective intracellular exposure to bioactive propolis constituents, thereby amplifying their biological activity. This behavior likely reflects a combination of factors, including increased intracellular exposure to propolis constituents and the presence of lipid components that may enhance cellular uptake or membrane interactions at higher concentrations [49]. Comparable trends have been reported for other propolis-loaded nanosystems, although absolute viability thresholds vary widely across studies. Syaifie et al., reported lower HepG2 viability for a chitosan-based nanosystem loaded with Indonesian propolis at similar concentrations, highlighting the influence of extract composition and carrier chemistry on cellular response [50]. On the other hand, Amin et al., showed that a nanoemulsion loaded with Egyptian propolis requires approximately 90.8 µg/mL to halve the viability of HepG2 [51]. In contrast, Tzankova et al. reported acceptable cell viability for both free propolis and propolis incorporated into a micellar system at concentrations exceeding 50 µg/mL in the same cell line [52]. Taken together, these observations underscore that the cytocompatibility of propolis-loaded nanosystems does not follow a universal trend but is strongly dependent on the chemical composition of the extract, the nature of the carrier, and the resulting interactions with cells. In this context, the present results indicate that NLC–Pe exhibits acceptable cytocompatibility at concentrations relevant for in vitro evaluation, while higher concentrations induce a predictable, dose-dependent reduction in viability. These findings support the suitability of the selected formulation for further mechanistic and biopharmaceutical investigations, while also highlighting the need for careful dose selection in future biological studies.

## 3. Materials and Methods

### 3.1. Materials

Gelucire^®^ 44/14 was donated by Gattefossé (Saint-Priest, France). Miglyol^®^ 812, Tween^®^ 80, dimethyl sulfoxide (DMSO, Molecular Biology grade), Folin–Ciocalteu reagent, sodium carbonate (Na_2_CO_3_), gallic acid, 2,2-diphenyl-1-picrylhydrazyl (DPPH•, ≥90%), ABTS Single Reagent, fluorescein, 2,2′-Azobis(2-amidinopropane) dihydrochloride (AAPH, 97%), sodium chloride (NaCl), L-α-lecithin (lecithin), pepsin, hydrochloric acid (HCl), sodium taurocholate, sodium hydroxide, acetic acid, sodium phosphate dibasic (Na_2_HPO_4_), 3-(4,5-dimethylthiazol-2-yl)-2,5-diphenyltetrazolium (MTT, ≥97.5%), and Amicon^®^ Ultra 10 kDa were purchased from Merck (Merck KGaA, Darmstadt, Germany). SnakeSkin^TM^ dialysis tubing (cellulose membrane, 10 kDa MWCO) was purchased from Thermo Fisher Scientific (Carlsbad, CA, USA). Chilean crude propolis samples were obtained from three locations in the Metropolitan Region of Chile: Peñaflor (33°37′00″ S, 70°55′00″ W), Pudahuel (33°26′42″33 S, 70°44′28″7 W), and Pirque (33°37′48″ S, 70°34′12″ W). Dulbecco’s Modified Eagle Medium (DMEM high-glucose; Cat# SH30243.02), phosphate-buffered saline (PBS, without calcium and magnesium; Cat# SH302560.1), fetal bovine serum (FBS; Cat# SV30160.03), and penicillin/streptomycin (Cat# SH40003.01) solution were obtained from Cytiva (Marlborough, MA, USA). Trypsin-EDTA solution (0.25%; Cat# 25200-072) was purchased from Thermo Fischer Scientific (Gibco, Waltham, MA, USA). Ultra-pure water (18.2 MΩ·cm) was produced using a Simplicity System from Millipore (Merck KGaA, Darmstadt, Germany). All materials were used as received without any further purification.

### 3.2. Methods

#### 3.2.1. Extraction and Dewaxing of Raw Propolis

Ethanolic propolis extracts (EPEs) were prepared according to the methodology described by Valenzuela-Barra et al., with minor modifications [53]. Briefly, raw propolis samples, previously cleaned to remove hive residues, foreign materials, and insects, were macerated in technical-grade ethanol (95%) at a 1:5 (*w*/*v*) ratio and maintained at 4–8 °C for approximately 24 h. Subsequently, the mixtures were heated in a thermostatically controlled water bath at 70 °C for 30 min and then returned to 4–8 °C for an additional 24 h. The resulting suspensions were then cold-filtered under reduced pressure to remove waxes. To enhance extraction efficiency, the temperature cycle and subsequent filtration step were repeated once. Combined filtrates were concentrated under reduced pressure using a rotary evaporator at 50 °C. The concentrated EPEs were dried in a vacuum oven at 45–50 °C for 72 h until constant weight was achieved. Finally, the dried extracts were stored protected from light until further use. Extraction yield was calculated based on the recovery of dry mass.

#### 3.2.2. Antioxidant Profile and Total Phenolic Content

The antioxidant profile of free propolis extracts and propolis-loaded NLC was evaluated using complementary spectrophotometric and fluorimetric assays in order to assess both reducing capacity and radical scavenging capacity. All determinations were performed using a microplate reader (BioTek Instruments, Inc., Winooski, VT, USA). Each analysis was conducted at least in triplicate.

Total phenolic content (TPC) was determined using a modified Folin–Ciocalteu colorimetric method [22]. Free propolis extracts were dissolved in ethanol, while propolis-loaded NLC were dispersed under mild agitation to obtain homogeneous suspensions. Aliquots of each sample were mixed with 10% Folin–Ciocalteu reagent and allowed to react for a short pre-incubation period (2 min at 40 °C). Subsequently, 5% Na_2_CO_3_ solution was added, and the reaction mixtures were incubated for 20 min at 40 °C, and absorbance was measured at 765 nm. Gallic acid was used as reference standard, and results were expressed as milligrams of gallic acid equivalents per gram of dry sample (mg GAE/g).

The free radical scavenging capacity against DPPH• was evaluated by determining the concentration required to inhibit 50% of the radical signal (SC_50_) [54]. Sample solutions or dispersions (50 µL) were mixed with 150 µL of ethanolic DPPH• solution (156 µM) and incubated in the dark at room temperature to allow reaction completion (30 min). Absorbance was recorded at 517 nm, and SC_50_ values were expressed as μg/mL.

The ABTS•^+^ scavenging capacity was assessed by determining the SC_50_ value following incubation of the samples with a pre-formed ABTS radical cation solution [55]. Sample aliquots (50 µL) were mixed with 150 µL of ABTS radical solution (169 µM) during a fixed incubation period (30 min) in the absence of light; then, absorbance was measured at 732 nm. The antioxidant capacity (SC_50_) was expressed as μg/mL.

The Ferric-Reducing Antioxidant Power (FRAP) assay was used to assess the reducing capacity of the samples based on the reduction of the Fe^3+^–TPTZ complex to its ferrous form [56]. Briefly, 10 µL of sample aliquots were mixed with freshly prepared FRAP reagent (290 µL) and incubated in the dark for 60 min. Absorbance was measured at 593 nm, and results were expressed as µmol Trolox equivalents per gram of sample (μmol Trolox/g).

The Oxygen Radical Absorbance (ORAC) assay was performed to evaluate peroxyl radical scavenging activity using fluorescein as a fluorescent probe [57]. To this, 45 µL of samples were pre-incubated with 175 µL of fluorescein solution (108 nM) at 37 °C for 30 min, followed by the addition of AAPH (18 mM) as a peroxyl radical generator. Fluorescence decay was monitored every 2 min during 2 h at excitation and emission wavelengths of 480 and 520 nm, respectively. Antioxidant capacity was quantified by calculating the area under the fluorescence decay curve and expressed as micromoles of Trolox equivalents per gram of sample (µmol Trolox/g).

#### 3.2.3. Fabrication of Nanostructured Lipid Carrier and Propolis Incorporation

Nanostructured lipid carriers (NLCs) were prepared using a slow-drip hot emulsification method, as previously described by our group, with minor adaptations [58]. The lipid phase consisted of Gelucire^®^ 44/14, Miglyol^®^ 812, and Tween^®^ 80, while ultrapure water (Milli Q^®^) was used as the aqueous phase. Briefly, Gelucire^®^ 44/14, Miglyol^®^ 812 and Tween^®^ 80 were combined and heated to 85 °C in a round-bottom flask under gentle stirring to obtain a homogeneous molten lipid phase. In parallel, the aqueous phase was heated to the same temperature. When both phases reached the temperature, the aqueous phase was slowly dripped onto the lipid phase under constant stirring (400 rpm). The mixture was cooled at 4 °C for at least 30 min without stirring to promote lipid solidification and nanoparticle formation.

For propolis incorporation, the ethanolic propolis extract (EPE) was added directly to the lipid phase prior to emulsification. The fabrication process was otherwise identical to that described above. The amount of propolis incorporated was selected according to the highest percentage of incorporation, and the resulting formulation was denominated NLC-P.

#### 3.2.4. Colloidal Characterization of NLC and NLC-P

The hydrodynamic diameter and polydispersity index (PdI) of NLC and NLC-P were evaluated by dynamic light scattering (DLS), while zeta potential was determined by laser Doppler electrophoresis using a Zetasizer Nano ZS90 (Malver Instruments, Malver, UK). Samples were diluted 1:10 in deionized water and analyzed at a wavelength of 633 nm with a detection angle of 173° and equilibration time of 120 s. Each reported value corresponds to the average of three independent measurements, each consisting of eleven determinations. The pH of the dispersion medium (deionized water) was not adjusted and was approximately 6.5 under the experimental conditions.

In addition, colloidal stability in the time was measured by three months at 4 °C and 25 °C. Hydrodynamic diameter, PdI and zeta potential were monitored at predefined time points and compared with freshly prepared formulations.

#### 3.2.5. Evaluation of Propolis Loading Capacity

The maximum loading capacity of the selected propolis in the NLC system was systematically evaluated by varying the propolis-to-lipid ratio. Propolis concentrations of 1.0, 0.6, 0.3, 0.15, and 0.07 g of propolis per 1 g of Gelucire^®^ 44/14 were investigated. To quantify the propolis incorporation, NLC-Pe formulations were subjected to ultrafiltration in 10,000 Da MWCO Amicon^®^ Ultra at 6900× *g* for 10 min. The ultrafiltrate containing the non-encapsulated fraction was collected and analyzed spectrophotometrically.

Propolis concentrations were determined by UV–Vis spectroscopy at 283 nm using a Genesys 180 UV–Vis spectrophotometer (Thermo Scientific, Waltham, MA, USA). Quantification was performed using a calibration curve constructed from serial dilutions of the same ethanolic propolis extract used for nanoparticle preparation [26]. Because propolis is a chemically complex natural extract composed of multiple phenolic and flavonoid constituents rather than a single defined compound, the analytical objective of this method was to estimate the total amount of extract present in the sample rather than quantify individual molecular species [59,60]. Accordingly, measured concentrations were expressed as propolis extract equivalents, determined by interpolation from the calibration curve.

The drug loading and incorporation efficiency percentages were determined according to the following Equation (1):

(1) $$\%\;Encapsulation\;efficiency\;\left(\%EE\right)=\frac{{Propolis}_{total}-{Propolis}_{free}}{{Propolis}_{total}}*100$$

where Propolis_total_ corresponds to the initial amount of propolis added during formulation and Propolis_free_ corresponds to the amount detected in the ultrafiltrate.

#### 3.2.6. In Vitro Release Study

Release studies were performed under gastrointestinally relevant residence times using both standard aqueous buffers and the bile-component-containing simulated media. Propolis release from the NLC formulation was determined using the dialysis bag method designed to monitor the fraction of propolis that becomes accessible to the surrounding medium. Briefly, SnakeSkin™ dialysis tubing (10 kDa MWCO) was preconditioned according to the manufacturer’s instructions and filled with a defined volume of NLC-Pe dispersion. The dialysis bags (3 mL) were immersed in 30 mL of release medium maintained at 37 ± 0.5 °C under continuous agitation (100 rpm) using an orbital incubator shaker (LabTech, LSI-3016R, Bucheon, Korea). At predetermined time points, aliquots (1.5 mL) were withdrawn from the external medium and immediately replaced with an equal volume of fresh medium to maintain sink conditions. Release experiments in standard aqueous buffers were performed at pH 1.2 and pH 6.8 for 2 h and 6 h, respectively, in accordance with gastrointestinally relevant residence times.

Propolis concentration in the collected samples was quantified by spectrophotometry at 283 nm as described above. Based on the measured concentration and the sampling volume, the cumulative amount of propolis extract equivalents released into the medium was calculated and expressed as µg of propolis extract equivalents. Results are therefore reported as cumulative apparent release over time. Because bile-component-containing media generate mixed colloidal structures capable of solubilizing lipophilic compounds, the measured values represent the analytically accessible fraction of propolis in the external medium, rather than complete depletion of propolis from the carrier or total nanoparticle disintegration. This distinction is well established for FaSSIF- and FeSSIF-type media, where solubilization and partitioning phenomena strongly depend on the colloidal phase behavior and analytical separation conditions [33].

#### 3.2.7. In Vitro Release Study in Simulated Gastrointestinal Media Containing Bile Components

To investigate the physicochemical response of propolis-loaded NLC under biorelevant conditions, release studies were conducted in simulated gastrointestinal media containing bile components at different pH values. A fasted-state simulated gastric fluid (FaSSGF, pH 1.6) was prepared using NaCl and lecithin (20 µM), supplemented with pepsin (0.1 g), and adjusted pH with HCl. Sodium taurocholate was included at a sub-millimolar concentration (80 µM) [61]. A fed-state simulated intestinal fluid (FeSSIF, pH 5.0) was prepared using sodium taurocholate (15 mM) and lecithin (3.75 mM), together with NaCl (11.87 g), NaOH (4.04 g), and acetic acid (8.65 g) to reach the target composition and pH. A fasted-state simulated intestinal fluid (FaSSIF, pH 6.5) was prepared using sodium taurocholate (3 mM) and lecithin (0.75 mM), with Na_2_HPO_4_ (3.438 g) and NaCl (6.18 g), adjusting pH with NaOH as required [62].

These media were selected because bile salts and phospholipids are known to form mixed colloidal assemblies that strongly modulate lipid solubilization, interfacial organization, and the apparent release behavior of lipid-based delivery systems [30].

It should be noted that no lipolytic enzymes were included in the simulated intestinal media. This deliberate choice was made to isolate the physicochemical contribution of bile components (bile salts and phospholipids) to carrier remodeling and payload accessibility. Enzyme-mediated lipolysis and fully dynamic digestion processes, which are known to further influence lipid-based systems, were therefore intentionally excluded and are considered beyond the scope of the present study.

#### 3.2.8. Morphologic Analysis Before and After Exposure to the Simulated Gastrointestinal Media

Transmission electron microscopy (TEM) was employed to qualitatively assess morphological changes in NLC–Pe before and after exposure to the simulated gastrointestinal media. TEM images were acquired using a LIBRA 120 transmission electron microscope (Zeiss, Jena, Germany), and all micrographs presented were obtained under identical imaging conditions. Freshly prepared NLC-Pe were used as controls, while treated samples were recovered after incubation under the corresponding conditions (FaSSGF pH 1.6, 2 h; FeSSIF pH 5.0, 6 h; FaSSIF pH 6.5, 6 h). TEM micrographs were analyzed qualitatively to evaluate particle integrity, size heterogeneity, aggregation behavior, and the formation of secondary lipid or mixed colloidal structures. TEM analysis was intended to provide structural insight into bile-component-induced remodeling rather than quantitative size determination.

#### 3.2.9. Cellular Viability

Cell viability of the developed formulations, free ethanolic propolis extract (EPE), unloaded NLC, and NLC-Pe, were evaluated in vitro using the colorimetric MTT assay. The human hepatocellular carcinoma cell line HepG2 (ATCC^®^ HB-806, Manassas, VA, USA), widely used as a model for hepatotoxicity and hepatic metabolism, was employed for this purpose [26]. HepG2 cells were cultured in DMEM with high glucose content, supplemented with 10% FBS and penicillin (100 µg/mL)–streptomycin (100 µg/mL). Cultures were maintained at 37 °C in a humidified atmosphere containing 5% CO_2_. Upon reaching approximately 90% confluence, cells were washed twice with 1× PBS and detached using 0.25% trypsin–EDTA. Cells were then seeded into 96-well plates at a density of 1 × 10^4^ cells per well in a final volume of 100 µL and incubated for 24 h to allow cell attachment. After the adhesion period, the culture medium was removed, and cells were treated with the different formulations diluted in supplemented culture medium. Final concentrations of 10, 20, 40, 80, and 100 µg/mL were evaluated at exposure times of 24 h and 48 h. At each time point, MTT solution was added to each well to achieve a final concentration of 0.5 mg/mL, and plates were incubated for 1.5 h at 37 °C. Absorbance was measured at 570 nm using a microplate reader (Synergy H1, BioTek, Winooski, VT, USA) with Gen5 software (v3.16) [63]. Cell viability was calculated as the percentage of absorbance relative to the negative control, according to the following equation:

(2) $$Cellular\;viability\;\left(\%\right)=\frac{\left(sample\;absorbance-blank(medium+EPE,\;NLC\;or\;NLC\text{-}Pe\right)}{Mean\;(control\;absorbance-\left(blanck\;medium\right))}*100$$

#### 3.2.10. Statistical Analysis

Statistical significance was determined at *p* < 0.05 using one-way or two-way ANOVA, as appropriate, followed by Tukey’s post hoc test, performed with GraphPad Prism version 8.0.1 (San Diego, CA, USA). Results are expressed as mean ± standard deviation (SD) from three independent experiments performed in triplicate.

## 4. Conclusions

In this study, an NLC based on Gelucire^®^ 44/14 was successfully developed as a delivery platform for a selected Chilean ethanolic propolis extract. The resulting nanosystem exhibited high encapsulation efficiency, narrow size distribution, and sustained physicochemical stability over time, supporting its suitability for the incorporation of chemically complex natural extracts.

Release experiments in simple aqueous buffers demonstrated strong retention of propolis within the lipid matrix, consistent with diffusion- and partition-controlled transport rather than rapid carrier disintegration. In contrast, exposure to simulated gastrointestinal media containing bile components induced pronounced pH- and composition-dependent effects. It is noteworthy that the extensive morphological remodeling observed by transmission electron microscopy did not translate into proportional increases in analytically accessible propolis, underscoring the distinction between structural alteration of the carrier and measurable release. In bile-rich media, propolis largely remained associated with mixed lipid-bile colloidal assemblies, whereas fasted-state intestinal conditions favored a higher accessible fraction without complete disruption of the nanosystem.

These findings highlight that apparent release from lipid-based nanocarriers under biorelevant conditions reflects complex partitioning, redistribution, and sequestration processes rather than a simple depletion of the payload. By deliberately excluding lipolytic enzymes, the present work isolates the physicochemical contribution of bile salts and phospholipids, providing mechanistic insights into early-stage carrier remodeling prior to enzymatic digestion. Preliminary cytocompatibility studies further indicated concentration-dependent effects that are consistent with previously reported propolis-loaded nanosystems.

Overall, this work demonstrates that nanostructured lipid carriers can effectively retain and modulate the accessibility of propolis under relevant gastrointestinal conditions, and that bile-induced structural changes do not necessarily imply increased release. The results contribute to a deeper physicochemical understanding of lipid-based delivery systems for complex natural products and establish a robust framework for future studies incorporating dynamic digestion models and absorption-related processes.

## Figures and Tables

**Table 1:** Antioxidant profile and total phenolic content of free propolis extracts from Peñaflor, Pirque, and Pudahuel, evaluated by DPPH•, ABTS•^+^, ORAC, FRAP, and total phenolic content (TPC) assays.

Sample	DPPH• ^a^	ABTS•^+ a^	ORAC ^b^	FRAP ^b^	TPC ^c^
Peñaflor	53.4 ± 0.02	2.6 ± 0.08	867.2 ± 4.02	222.9 ± 0.06	40.7 ± 0.02
Pirque	65.4 ± 0.02	14.2 ± 0.04	472.3 ± 1.17	215.9 ± 0.02	36.6 ± 0.02
Pudahuel	68.3 ± 0.02	17.9 ± 0.01	321.6 ± 2.95	204.4 ± 0.02	35.4 ± 0.03

^a^ Antiradical DPPH and ABTS activities are expressed as μg/mL; ^b^ Expressed as μmol Trolox/g Extract; ^c^ Total phenolic content (TPC) expressed as mg gallic acid equivalent GAE/g extract.

**Table 2:** Physicochemical characterization of NLC and NLC-Pe, showing hydrodynamic diameter (nm ± SD), polydispersity index (± SD) and zeta potential (mV ± SD) (*n* = 3).

Nanosystem	Hydrodynamic Diameter (nm ± SD)	Polydispersity Index (±SD)	Zeta Potential (mV ± SD)
NLC	150.4 ± 3.2	0.11 ± 0.01	−10.3 ± 0.5
NLC-Pe	206.5 ± 1.9	0.12 ± 0.01	−8.0 ± 0.4

## Data Availability

The manuscript and Supplementary Materials contain the reported data. Additional relevant data can be obtained upon request from the corresponding author.

## Supplementary Materials

The following supporting information can be downloaded at: https://www.mdpi.com/article/10.3390/molecules31061028/s1, Table S1: Antioxidant profile and total phenolic content of NLC loaded with propolis extracts from Peñaflor, Pirque, and Pudahuel, evaluated by DPPH•, ABTS•+, ORAC, FRAP, and total phenolic content (TPC) assays.
