# Supplementary material for: Nanostructured Lipid Carriers as Physicochemical Modulators of Complex Natural Extracts: Release Behavior and Bile-Induced Remodeling in Biorelevant Media
Source: Molecules. 2026 Mar 19;31(6):1028. doi: 10.3390/molecules31061028 (PMC13029395; doi:10.3390/molecules31061028)
Supplement: Supplementary file 1 [file molecules-31-01028-s001.zip › molecules-4183043-supplementary.pdf]

Nanostructured Lipid Carriers as Physicochemical Modulators of Complex Natural Extracts: Release Behavior and Bile-Induced Remodeling in Biorelevant Media

Javiera Carrasco-Rojas [1], Javiera Solas-Soto [2], Rubén Veas-Albornoz [2], Carlos F. Lagos [2,3], Mario J. Simirgiotis [4], Francisco Arriagada [1] and Andrea C. Ortiz [2,*]

[1] Departamento de Ciencias y Tecnología Farmacéutica, Facultad de Ciencias Químicas y Farmacéuticas, Universidad de Chile, Santiago 8380494, Chile;
[2] Escuela de Química y Farmacia, Facultad de Ciencias, Universidad San Sebastián, Lota 2465, Santiago 7510157, Chile
[3] Centro Basal Ciencia & Vida, Fundación Ciencia & Vida, Av. del Valle Norte 725, Santiago 8580702, Chile
[4] Instituto de Farmacia, Facultad de Ciencias, Universidad Austral de Chile, Campus Isla Teja, Valdivia 5090000, Chile
* Correspondence: andrea.ortizo@uss.cl

Table S1. Antioxidant profile and total phenolic content of NLC loaded with propolis extracts from Peñaflor, Pirque, and Pudahuel, evaluated by DPPH•, ABTS•+, ORAC, FRAP, and total phenolic content (TPC) assays.

Sample	DPPH[a]	ABTS[a]	ORAC[b]	FRAP[b]	TPC[c]
NLC-Peñaflor	322.1 ± 0.03	253.9 ± 0.01	472.1 ± 2.04	24.8 ± 0.02	4.9 ± 0.01
NLC-Pirque	475.4 ± 0.01	351.5 ± 0.02	291.8 ± 3.05	11.9 ± 0.01	2.5 ± 0.01
NLC-Pudahuel	480.7 ± 0.04	406.5 ± 0.02	250.9 ± 1.72	11.6 ± 0.04	1.1 ± 0.02

[a]Antiradical DPPH and ABTS activities are expressed as μg/mL; [b]Expressed as μmol Trolox/g Extract; [c]Total phenolic content (TPC) expressed as mg gallic acid equivalent GAE/ g extract.
